# Clinical outcomes of COPD associated with cigarette smoke, biomass smoke, and combined exposure: A retrospective observational study

**DOI:** 10.18332/tid/231161

**Published:** 2026-10-23

**Authors:** Aycan Yüksel, Dorina Esendağlı, Polina Nezalzova, Gaye Ulubay

**Affiliations:** 1 Department of Respiratory Medicine, Faculty of Medicine, TOBB University of Economics and Technology, Ankara, Türkiye; 2 Department of Respiratory Medicine, Başkent University Faculty of Medicine, Ankara, Türkiye

**Keywords:** biomass, chronic obstructive pulmonary disease, exacerbations, FEV1, tobacco

## Abstract

**INTRODUCTION:**

Chronic obstructive pulmonary disease (COPD) related to combined exposure to cigarette and biomass smoke is insufficiently characterized. The aim of this study was to compare exacerbations and chronic respiratory failure (CRF) across smoking-associated, biomass-associated, and combined exposure COPD.

**METHODS:**

In this retrospective, multicenter, observational study, 319 patients with stable COPD were classified into three groups based on exposure history: smoking-associated (n=159), biomass-associated (n=80), and combined cigarette and biomass exposure (n=80). Smoking and biomass exposure histories were retrospectively obtained from medical records. Cigarette exposure was quantified in pack-years, biomass exposure was assessed according to the documented type and duration of household fuel use, and patients with both exposures were classified as the combined-exposure group. Exacerbation frequency, CRF, and pulmonary function tests were evaluated. Multivariable models adjusted for age, sex, cardiac diseases, sarcopenia, and lung cancer were used to assess the associations of exposure group with annual exacerbation frequency and CRF.

**RESULTS:**

Patients with combined exposure were younger (59.22 ± 10.82 years) compared with those in the smoking-associated (61.37 ± 11.04 years) and biomass-associated groups (63.45 ± 11.92 years, p=0.015). The combined exposure group had lower FEV_1_ (% predicted: 48.27 ± 10.14 vs 55.28 ± 17.61 and 55.62 ± 11.29; p=0.003) and higher dyspnea scores (mMRC: 2.18 vs 1.39 and 1.88; p<0.001). Exacerbation burden was significantly greater in the combined exposure group, including higher annual exacerbation rates (2.18 ± 0.82 vs 2.08 ± 0.22 and 1.88 ± 0.32; p=0.041) and severe exacerbations (1.78 ± 0.35 vs 1.39 ± 0.18 and 1.20 ± 0.19; p<0.001). CRF was more frequent in the combined-exposure group (26.2% vs 17.0% and 11.2%; p=0.028). In adjusted analyses, combined exposure was independently associated with increased risk of severe exacerbations compared with cigarette smoking alone (AOR=1.89; 95% CI: 1.22–2.96).

**CONCLUSIONS:**

Findings of this study suggest that combined exposure to cigarette and biomass smoke is potentially associated with earlier COPD presentation, increased exacerbation frequency and lower FEV_1_.

## INTRODUCTION

Chronic obstructive pulmonary disease (COPD) is a leading cause of morbidity and mortality worldwide and represents a major public health burden^1^. It is characterized by persistent airflow limitation, chronic airway inflammation, and progressive respiratory symptoms, often accompanied by systemic manifestations and comorbidities^1^. Tobacco smoking is the most widely recognized risk factor for COPD; however, non-smoking-related exposures play a substantial role in disease development, particularly in low- and middle-income countries (LMICs)^1^.

Air pollution is an important environmental risk factor for COPD, particularly in regions where exposure to household air pollution remains widespread. Household air pollution resulting from biomass fuel combustion is one of the most important non-tobacco risk factors for COPD^1,2^. Biomass fuels such as wood, animal dung, crop residues, and coal are commonly used for cooking and heating, especially in rural areas^1,3^. Inadequate ventilation and inefficient stoves can result in high indoor concentrations of particulate matter and toxic combustion products, including carbon monoxide, volatile organic compounds, aldehydes, and polycyclic aromatic hydrocarbons, several of which are also constituents of tobacco smoke^4^. Globally, nearly three billion people rely on biomass fuels for daily household needs, placing a large population at risk for chronic respiratory diseases^5^.

Biomass-associated COPD has been reported to exhibit different clinical, functional, and radiological characteristics compared with smoking-associated COPD^2,3,6-9^. Previous studies suggest that biomass exposure is more frequently associated with chronic bronchitis and lower emphysema burden, whereas tobacco-associated COPD is more commonly characterized by emphysema^2,7,8,10^. Moreover, biomass-associated COPD disproportionately affects women living in rural areas, while cigarette-associated COPD predominantly affects men^1^. Despite these differences, whether biomass-associated COPD represents a different phenotype or merely an alternative exposure pathway leading to a similar disease spectrum remains a matter of debate^6^. Additionally, many patients, particularly in developing countries, are exposed to both tobacco smoke and biomass smoke over their lifetime^11,12^. However, patients with dual exposure have often been excluded from previous comparative studies. Despite this common scenario, COPD associated with combined exposure has been insufficiently studied and is often excluded from comparative analyses. Concomitant exposure to tobacco and biomass smoke may be clinically relevant because both sources may contribute to cumulative inhalational injury over time^4,11^.

Assessment of COPD prognosis depends on multiple parameters, including symptom burden, spirometric impairment, exacerbation frequency, and the presence of complications such as pulmonary hypertension (PH) and chronic respiratory failure (CRF)^1^. Acute exacerbations are particularly important, as they accelerate lung function decline, worsen quality of life, and increase mortality^13-15^. While several studies have compared smoking-associated and biomass-associated COPD, it remains unclear whether combined exposure is associated with more frequent exacerbations, lower FEV_1_, and a poorer clinical prognosis than either exposure alone^7-9^.

The aim of this multicenter retrospective observational study was to compare annual exacerbation frequency and the prevalence of major complications, including pulmonary hypertension and chronic respiratory failure, among patients with smoking-associated COPD, biomass-associated COPD, and COPD associated with combined cigarette and biomass smoke exposure, who were evaluated at two tertiary-care centers in Ankara, Türkiye, between 1 January 2024 and 31 August 2025.

## METHODS

### Study design and setting

This multicenter, retrospective, observational study included patients with stable COPD. Consecutive COPD patients attending outpatient Pulmonology clinics of two tertiary-care centers (Başkent University Faculty of Medicine and TOBB University of Economics and Technology Faculty of Medicine) between 1 January 2024 and 31 August 2025 were screened for eligibility. Of the 413 patients with stable COPD, 94 were excluded, and the remaining 319 patients were classified as having smoking-associated COPD (n=159), biomass-associated COPD (n=80), or COPD associated with combined exposure (n=80), as shown in Figure 1.

**Figure 1:**
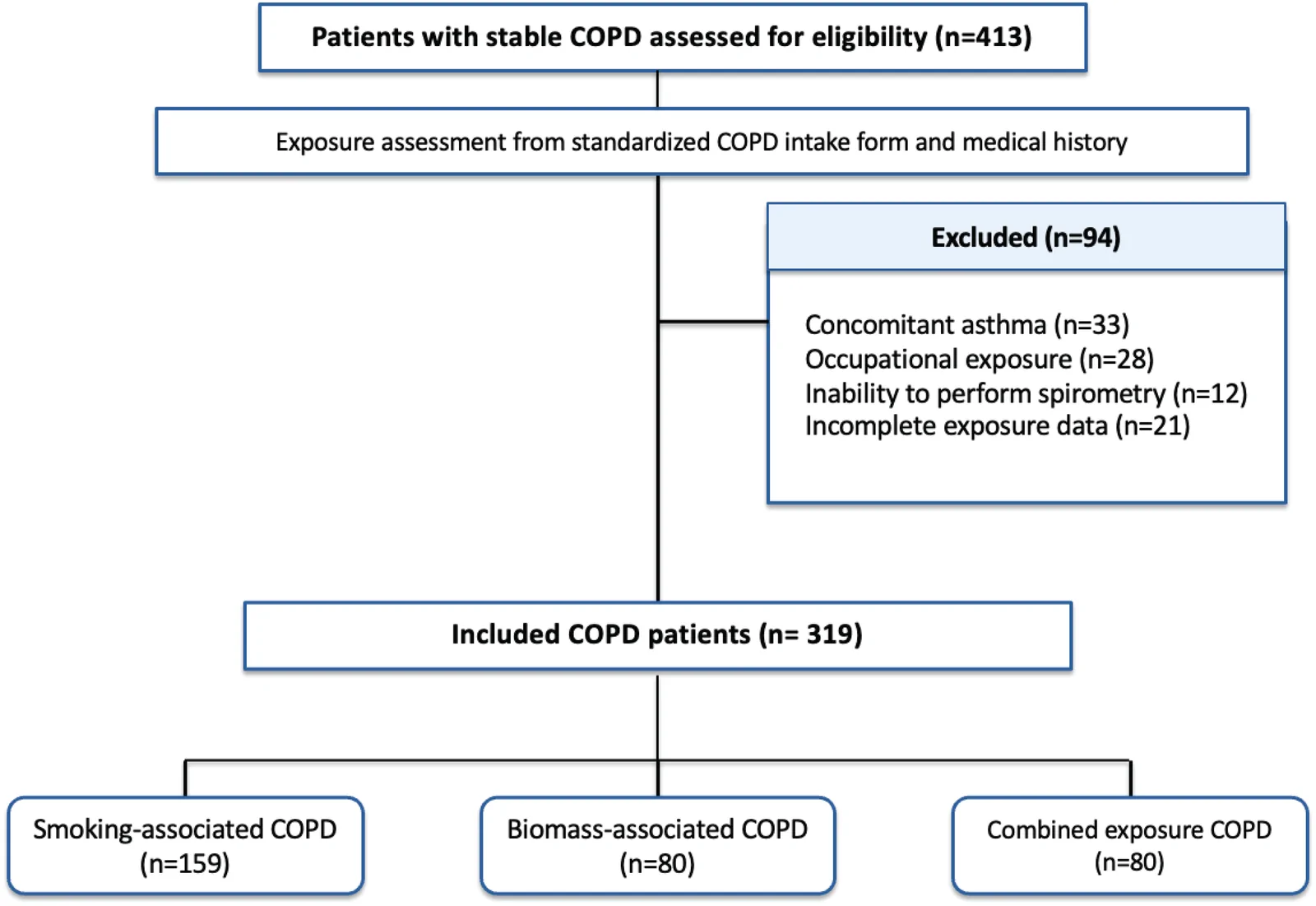
Study flow diagram of patients with COPD screened at two tertiary-care centers in Ankara, Türkiye, 1 January 2024 – 31 August 2025 (N=413)

### Study population

COPD patients diagnosed with a lower FEV_1_/FVC ratio than 0.7 were included^1^. Patients meeting the following criteria were included in the study: age ≥40 years, stable COPD, spirometry-confirmed diagnosis of COPD, and COPD related to tobacco smoke and/or biomass smoke exposure. Exclusion criteria were as follows: pregnancy, occupational exposure to dusts or harmful gases, asthma, and inability to perform acceptable pulmonary function testing. Included patients were classified into three groups based on their etiotypes: 1) Smoking-associated COPD (patients with a history of cigarette smoking and no biomass exposure); 2) Biomass-associated COPD (patients with a history of biomass exposure and no cigarette smoking); and 3) Combined exposure-associated COPD, i.e. patients with a history of both cigarette and biomass smoke exposure.

### Exposure assessment

At both participating centers, all patients undergo a standardized medical history assessment at the time of COPD diagnosis. This structured form systematically records cigarette smoking, biomass smoke exposure, occupational and environmental exposures, and previous respiratory infections. For the present study, exposure data were retrospectively extracted from these standardized clinical records. Cigarette smoking history included smoking status and cumulative exposure expressed in pack-years. Biomass smoke exposure was characterized according to its purpose, including cooking, heating, or both; the type of fuel used, such as wood, sawdust, animal dung, crop residues, or coal; total duration of exposure in years; frequency of exposure, recorded as the number of days per week; and daily exposure, recorded as the average number of hours per day. Biomass smoke exposure was quantified in hour-years by multiplying the average daily duration of exposure in hours by the total duration of exposure in years. Information regarding cooking and heating practices and household ventilation conditions was also reviewed when available. Patients were classified into smoking-only, biomass-only, or combined-exposure groups according to the documented exposure history.

### Outcome definitions

An acute exacerbation of COPD was defined as an acute worsening of respiratory symptoms requiring treatment with systemic corticosteroids, antibiotics, emergency department visits, or hospitalization^1^. An exacerbation requiring hospitalization and/or leading to acute respiratory failure was defined as a severe exacerbation^1^. Patients were classified into GOLD groups A, B, and E according to symptom burden and exacerbation history: group A included patients with low symptom burden (mMRC score <2 or COPD Assessment Test [CAT] score <10) and no exacerbations requiring hospitalization, group B included patients with greater symptom burden (mMRC score ≥2 or CAT score ≥10) but without a high exacerbation risk, and group E included patients with a history of ≥2 moderate exacerbations or ≥1 exacerbation requiring hospitalization during the previous year, in accordance with GOLD recommendations^1^. Chronic respiratory failure (CRF) was defined based on documented hypoxemia (PaO_2_ <60 mmHg) and/or daytime hypercapnia (PaCO_2_ >53 mmHg) requiring long-term oxygen therapy (LTOT) and/or non-invasive mechanical ventilation (NIV)^1,16^. According to GOLD recommendations, LTOT is indicated in patients with severe chronic resting hypoxemia (PaO_2_ ≤55 mmHg, or <60 mmHg in the presence of cor pulmonale or secondary polycythemia), whereas long-term NIV is considered in patients with severe chronic hypercapnia (PaCO_2_ >53 mmHg), particularly those with a history of hospitalization for acute respiratory failure^1^.

### Data collection

The number of COPD exacerbations within the previous year was recorded. COPD exacerbations during the preceding 12 months were identified retrospectively from outpatient, emergency department, hospitalization, and prescription records. Pulmonary function tests (PFTs) including forced expiratory volume in one second (FEV_1_), forced vital capacity (FVC), FEV_1_/FVC ratio, and forced expiratory flow between 25% and 75% of FVC (FEF_25-75_) were recorded as absolute values and percentages of predicted values. Radiological images, including chest X-ray and thoracic computed tomography (if performed), were screened for the presence of emphysema. Disease severity groups and clinical phenotypes were determined based on exacerbation frequency, symptom scores, and spirometric findings. The presence of pulmonary hypertension was assessed using echocardiography.

### Ethics

This study was conducted in accordance with the Declaration of Helsinki and approved by the Ethics Committee of Başkent University Faculty of Medicine (Approval number: KA25/317; Date: 30 September 2025). Due to the retrospective design of the study, written informed consent was not required.

### Potential covariates

Potential covariates for the multivariable logistic regression models were first evaluated using univariable analyses. Candidate variables included major comorbidity domains, including cardiac, psychiatric, metabolic conditions, and sarcopenia, as well as lung cancer and FEV_1_. Variables meeting the predefined criterion of p<0.10 were considered for inclusion in the multivariable logistic regression models, while age and sex were retained based on their clinical relevance. Based on the univariable analyses, age, sex, cardiac comorbidity, sarcopenia, and lung cancer were included in the final multivariable models. Coronary artery disease, heart failure, and cardiac arrhythmias were combined into a single composite variable defined as cardiac comorbidity to reduce potential collinearity and model overparameterization.

### Statistical analysis

Statistical analyses were performed using SPSS version 22.0 (SPSS Inc., Chicago, IL, USA). *A priori* sample size calculation was performed using G*Power for the primary outcome of annual exacerbation frequency. For a one-way analysis of variance (ANOVA) comparing three exposure groups, assuming a medium effect size (Cohen’s f=0.25), a two-sided α level of 0.05, and 90% power, a minimum total sample size of 206 patients (69 patients per group) was required. Continuous variables are presented as mean and standard deviation (SD) if normally distributed, and as median and interquartile range (IQR) if not normally distributed. Normality of continuous variables was assessed using the Shapiro–Wilk test and visual inspection of histograms and Q–Q plots. Categorical variables were expressed as frequencies and percentages (%). No missing data were present for the variables included in the main analyses among the final study population. Twenty-one patients with incomplete exposure data were excluded before group classification, as shown in Figure 1. Comparisons among the three exposure groups were performed using one-way ANOVA or the Kruskal–Wallis test for continuous variables. *Post hoc* pairwise comparisons were conducted with Bonferroni correction. Categorical variables were compared using the chi-squared test or Fisher’s exact test. Multivariable models were adjusted for age, sex, and comorbidity burden. Multicollinearity among candidate covariates was assessed before multivariable modeling. Coronary artery disease, heart failure, and cardiac arrhythmias were combined into a single composite variable, cardiac comorbidity, to reduce potential collinearity and model overparameterization. No problematic multicollinearity was identified in the final models. A two-sided p<0.05 was considered statistically significant.

## RESULTS

### Study population and demographic characteristics

A total of 319 patients with stable COPD were included in the study. Patients were classified into three groups: smoking-associated COPD (n=159), biomass-associated COPD (n=80), and combined cigarette and biomass exposure-associated COPD (n=80). Baseline demographic and clinical characteristics of the study population are summarized in Table 1. Patients in the combined exposure COPD group were younger compared to those in the smoking- and biomass-associated COPD groups (p=0.015). There was a significant difference in sex distribution, with women comprising 87.5% of the biomass-associated group, 66.3% of the combined exposure group, and 26.4% of the smoking-associated group (p<0.001). Body mass index (BMI, kg/m^2^) was significantly higher in biomass-associated (27.2 ± 0.44) and combined exposure (27.4 ± 0.35) patients compared with smoking-related COPD (24.4 ± 0.26; p<0.001). The biomass exposure duration was similar between the two groups, whereas the type of biomass exposure was significantly different (p<0.001): biomass-associated COPD was predominantly related to heating exposure, while combined exposure COPD was mainly associated with both cooking and heating (Table 1). Charlson comorbidity index (CCI) was highest in the combined exposure COPD group (5.21 ± 2.06), compared with the biomass-associated (3.52 ± 1.98) and smoking-associated groups (3.21 ± 1.62). Ischemic heart disease was most prevalent in the smoking-associated group (64.2%), followed by the combined exposure (53.7%) and biomass groups (35.0%). Depression/anxiety, lung cancer, sarcopenia, and bronchiectasis were more common in the combined exposure group compared to the smoking and biomass groups; whereas hypertension, diabetes mellitus, arrhythmia, and osteoporosis showed no statistically significant differences across the groups (Table 1).

**Table 1:** Demographic characteristics of patients with COPD from two tertiary-care centers in Ankara, Türkiye, 1 January 2024 – 31 August 2025 (N=319)

*Characteristics*	*Smoking-associated COPD (N=159)* *n (%)*	*Biomass-associated COPD (N=80)* *n (%)*	*Combined exposure COPD (N=80)* *n (%)*	*p*
Age (years), mean ± SD	61.37 ± 11.04	63.45 ± 11.92	59.22 ± 10.82	0.015*
Women	42 (26.4)	70 (87.5)	53 (66.3)	<0.001*
BMI, mean ± SD	24.4 ± 0.26	27.2 ± 0.44	27.4 ± 0.35	<0.001*
**Smoking status^a^**				0.821
Current smoker	54 (34)		26 (32.5)	
Ex-smoker	105 (66)		54 (67.5)	
**Pack-years^a^,** mean ± SD	32.1 ± 8.9		31.7 ± 3.4	0.618
**Biomass exposure setting^b^**				<0.001*
Cooking only		12 (15)	15 (18.7)	
Heating only		62 (77.5)	13 (16.3)	
Cooking + Heating		6 (7.5)	52 (65)	
**Years of biomass exposure^b^,** mean ± SD		31.2 ± 2.8	30.8 ± 5.7	0.574
**Duration of biomass exposure^b^** (hour-years), mean ± SD		63.7 ± 8.3	62.9 ± 6.9	0.508
**Health status**				
CCI, mean ± SD	3.21 ± 1.62	3.52 ± 1.98	5.21 ± 2.06	<0.001*
Hypertension	92 (57.8)	42 (52.5)	45 (56.3)	0.73
Diabetes	72 (45.3)	47 (58.8)	38 (47.5)	0.14
Ischemic heart disease	102 (64.2)	28 (35)	43 (53.7)	<0.001*
Arrhythmia	43 (27)	23 (28.7)	28 (35)	0.44
Depression/Anxiety	28 (17.6)	13 (16.2)	33 (41.2)	<0.001*
Osteoporosis	33 (20.8)	18 (22.5)	19 (23.7)	0.86
Sarcopenia	46 (28.9)	23 (28.7)	35 (43.7)	0.049*
Lung cancer	28 (17.6)	7 (8.7)	21 (26.2)	0.015*

a Indicates a statistical comparison performed only between the smoking-associated COPD and combined-exposure groups.

b Indicates a statistical comparison performed only between the biomass-associated COPD and combined-exposure groups. BMI: body mass index (kg/m^2^). CCI: Charlson comorbidity index. COPD: Chronic obstructive pulmonary disease.

* A p-value <0.05. considered statistically significant.

### Clinical characteristics and disease severity

Pulmonary function tests and radiological findings are shown in Table 2. The combined exposure COPD group had lower FEV_1_ compared to the smoking- and biomass-associated groups (48.27 ± 10.14 vs 55.28 ± 17.61 and 55.62 ± 11.29, respectively; p=0.003). mMRC score was significantly higher in the combined exposure group (2.18 ± 0.78) compared to the biomass (1.88 ± 0.82) and smoking groups (1.39 ± 0.22; p<0.001). Emphysema was more frequent in the smoking-associated group (20.8%) compared to the biomass (6.2%) and combined groups (15.0%; p=0.006).

**Table 2:** Pulmonary function tests and clinical characteristics of patients with COPD from two tertiary-care centers in Ankara, Türkiye, 1 January 2024 – 31 August 2025 (N=319)

	*Smoking-associated COPD (N=159)* *Mean ± SD*	*Biomass-associated COPD (N=80)* *Mean ± SD*	*Combined exposure COPD (N=80)* *Mean ± SD*	*p*
**FEV_1_** (pred %)	55.28 ± 17.61	55.62 ± 11.29	48.27 ± 10.14	0.003*
**FVC** (pred %)	88.87 ± 9.78	85.93 ± 12.31	83.79 ± 10.23	0.021*
**FEV_1_/FVC** (%)	62.05 ± 15.5	59.27 ± 15.84	60.75 ± 13.87	0.38
**FEF_25-75_** (pred %)	44.34 ± 8.24	43.5 ± 6.85	42.68 ± 5.67	0.27
**mMRC score**	1.39 ± 0.22	1.88 ± 0.82	2.18 ± 0.78	<0.001*
**GOLD group**, n (%)				0.15
A	39 (24.5)	21 (26.2)	12 (15)	
B	54 (34)	29 (36.2)	33 (41.2)	
E	66 (41.5)	30 (37.5)	35 (43.7)	
**Emphysema**, n (%)	33 (20.8)	5 (6.2)	12 (15)	0.006*
**Bronchiectasis**, n (%)	33 (20.8)	35 (43.7)	42 (52.5)	<0.001*

GOLD: Global Initiative for Chronic Obstructive Lung Disease. FEV_1_: forced expiratory volume in 1 second. FVC: forced vital capacity. FEF_25-75_: forced expiratory flow between 25% and 75% of FVC. mMRC: modified Medical Research Council dyspnea scale.

* A p<0.05 considered statistically significant.

### Clinical outcomes

Annual exacerbation rates were higher in the combined group compared to smoking and biomass groups (2.18 ± 0.82 vs 2.08 ± 0.22 and 1.88 ± 0.32, respectively; p=0.041). Combined exposure COPD group had more severe exacerbations (1.78 ± 0.35 vs 1.39 ± 0.18 and 1.20 ± 0.19, respectively; p<0.001) and exacerbations requiring mechanical ventilation and/or intensive care unit (ICU) admission (1.01 ± 0.29 vs 0.39 ± 0.14 and 0.35 ± 0.15, respectively; p<0.001) (Table 3). CRF was more frequent in the combined group (Figure 2), reflected by higher rates of long-term oxygen therapy use (26.2% vs 17.0% and 11.2%; p=0.028), while home non-invasive ventilation use was similar between groups (Table 3). Pulmonary hypertension was also significantly more common in the combined exposure group (22.5%) compared to smoking (10.1%) and biomass groups (8.75%; p=0.021) (Table 3). After adjusted analysis, the odds of severe exacerbations were significantly higher in the combined exposure group than in the smoking-associated COPD group, which was used as the reference category (AOR=1.89; 95% CI: 1.22–2.96, p=0.004) (Table 4). Similarly, pulmonary hypertension was significantly more frequent in patients with combined exposure than in the smoking-associated COPD group (AOR=2.57; 95% CI: 1.28–5.15, p=0.008) (Table 4).

**Table 3:** Clinical outcomes and complications among patients with COPD from two tertiary-care centers in Ankara, Türkiye, 1 January 2024 – 31 August 2025 (N=319)

	*Smoking-associated COPD (N=159)* *Mean ± SD*	*Biomass-associated COPD (N=80)* *Mean ± SD*	*Combined exposure COPD (N=80)* *Mean ± SD*	*p*
**Annual exacerbations**	2.08 ± 0.22	1.88 ± 0.32	2.18 ± 0.82	0.041*
**Severe exacerbations**	1.39 ± 0.18	1.2 ± 0.19	1.78 ± 0.35	<0.001*
**Exacerbations requiring MV and/or ICU**	0.39 ± 0.14	0.35 ± 0.15	1.01 ± 0.29	<0.001*
**Chronic respiratory failure**, n (%)				
LTOT use	27 (17)	9 (11.2)	21 (26.2)	0.028*
H-NIV use	19 (11.9)	6 (7.5)	9 (11.2)	0.54
**Pulmonary hypertension**, n (%)	16 (10.1)	7 (8.75)	18 (22.5)	0.021*

H-NIV: home non-invasive ventilation. ICU: Intensive Care Unit. LTOT: long-term oxygen therapy. MV: mechanical ventilation.

* A p-value <0.05 considered statistically significant.

**Table 4:** Multivariable logistic regression analyses for severe exacerbations and pulmonary hypertension among patients with COPD from two tertiary-care centers in Ankara, Türkiye, 1 January 2024 – 31 August 2025 (N=319)

*Outcome*	*Variable (Exposure group)*	*AOR*	*95% CI*	*p*
Severe exacerbation	Smoking-associated COPD (ref.)	1.00		
	Biomass-associated COPD	1.09	0.87–1.22	0.678
	Combined exposure	1.89	1.22–2.96	0.004*
	Cardiac comorbidity	1.7	1.1–2.61	0.017*
	Sarcopenia	1.56	1.01–2.42	0.046*
	Lung cancer	1.48	1.12–3.05	0.028*
Pulmonary hypertension	Smoking-associated COPD (ref.)	1.00		
	Biomass-associated COPD	1.43	0.76–2.68	0.267
	Combined exposure	2.57	1.28–5.15	0.008*
	Cardiac comorbidity	2.16	1.26–3.72	0.005*
	Sarcopenia	1.91	1.07–3.40	0.028*
	Lung cancer	2.62	1.13–6.09	0.025*
***Model-fit statistics***	***Severe exacerbation model***		***Pulmonary hypertension model***	
Omnibus χ²	28.4		31.7	
Omnibus p	<0.001		<0.001	
-2 Log likelihood	356.8		271.5	
Nagelkerke R²	0.18		0.24	
Hosmer–Lemeshow χ²	6.12		5.48	
Hosmer–Lemeshow p	0.634		0.705	

AOR: adjusted odds ratio. CI: confidence interval. COPD: chronic obstructive pulmonary disease. Smoking-associated COPD was used as the reference exposure category. Cardiac comorbidity comprised coronary artery disease, heart failure, and cardiac arrhythmias.

**Figure 2:**
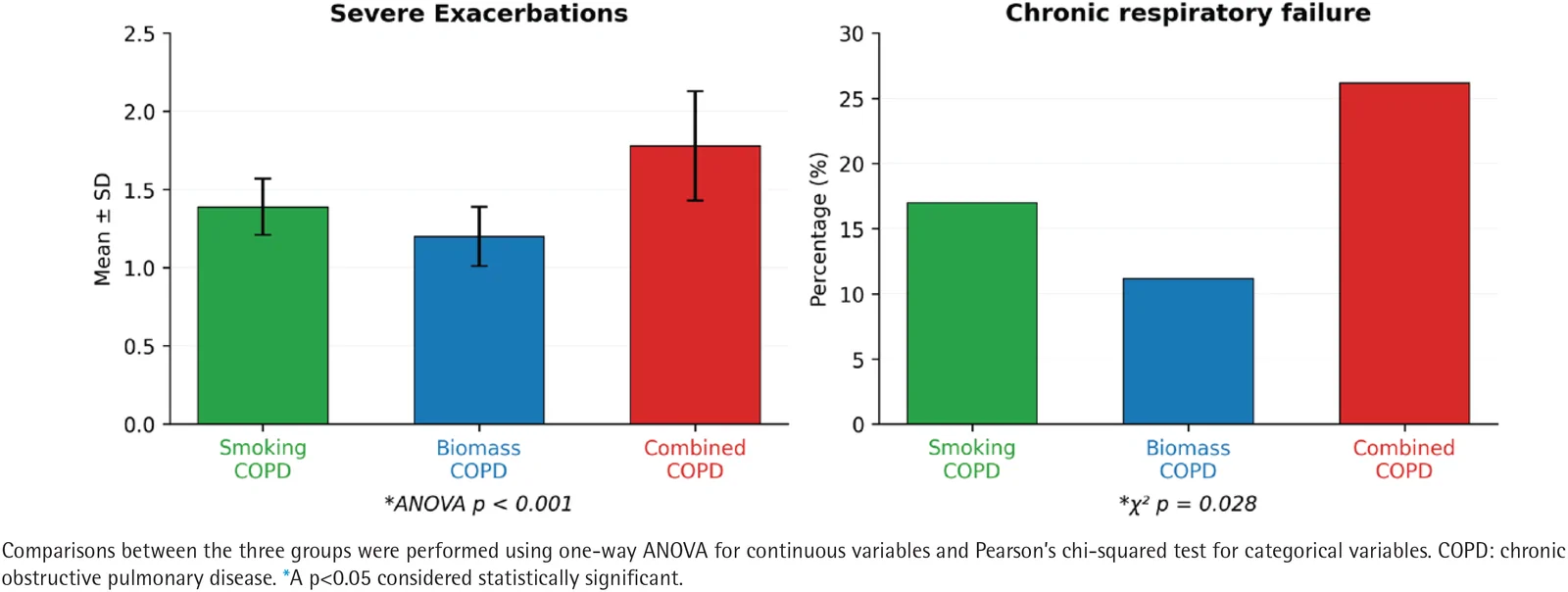
Clinical outcomes in exposure groups among patients with COPD from two tertiary-care centers in Ankara, Türkiye, 1 January 2024 – 31 August 2025 (N=319)

## DISCUSSION

In this retrospective observational study, combined exposure to cigarette and biomass smoke was associated with less favorable clinical outcomes, particularly exacerbations and pulmonary hypertension, than either exposure alone, despite the combined-exposure group being younger. Biomass-associated COPD was predominantly observed in women and was largely linked to heating exposure, whereas combined exposure typically reflected both cooking and heating. The combined-exposure group showed a higher comorbidity burden, lower FEV_1_, higher dyspnea scores, and a higher prevalence of bronchiectasis. These differences translated into more frequent and more severe exacerbations and greater ICU/mechanical ventilation burden. When taken together, the results support the concept that dual exposure (biomass plus tobacco) may accelerate clinical symptoms and worsen the course of the disease.

A key signal in this cohort is the ‘earlier’ presentation among patients with combined exposure. The combined-exposure group was younger than both the smoking-associated and biomass-associated groups yet had worse FEV_1_, higher dyspnea score and comorbidity burden, reflected by the highest CCI score. This finding may indicate an association between cumulative exposure to both cigarette and biomass smoke and an earlier clinical manifestation of COPD, although causality cannot be inferred from the present observational study. Multimorbidity is common in COPD; sarcopenia and depression/anxiety should be actively sought because they independently affect hospitalizations and mortality^17^. Sarcopenia is increasingly conceptualized as a measurable, modifiable ‘treatable trait’ in COPD and is linked with worse outcomes^18^. Depression has also been linked to exacerbation propensity: in a prospective cohort, frequent exacerbators had higher baseline depression scores than infrequent exacerbators, and depression worsened during exacerbations^17^. A cross-sectional study^19^ reported higher depression prevalence in biomass-exposed versus tobacco-exposed COPD (41.2% vs 27.7%) after adjustment for age and FEV_1_. In the current study, sarcopenia and depression/anxiety were similar in biomass- and cigarette-associated COPD but were substantially higher in combined exposure, which may contribute to worse functional reserve and higher exacerbation vulnerability. Nevertheless, combined tobacco and biomass exposure has rarely been characterized as a distinct COPD etiotype, and adjusted exposure-stratified estimates for these comorbidities are largely absent; residual confounding (including disease severity, socioeconomic factors, and activity limitation) and potential reverse causality remain important considerations.

Exposure histories also separated the groups in a direction consistent with known epidemiology. Women predominated in the biomass group (87.5%) and remained a majority in the combined-exposure group (66.3%), whereas they were a minority in the smoking-associated group (26.4%). The predominance of women in biomass-associated COPD in this cohort aligns with global and clinical observations that household air pollution, particularly from cooking and heating with solid fuels, disproportionately affects women, especially in LMICs^3,10^. Emphysema was most frequent in smoking-associated COPD, consistent with an emphysema-predominant pattern, whereas bronchiectasis was more common in combined exposure and biomass-associated COPD groups. Previous literature suggests that biomass-associated COPD is often airway-predominant, with comparatively less emphysema and more air trapping/airway thickening than tobacco-related COPD. In a CT-based analysis, biomass smoke exposure was associated with less emphysema but more air trapping than tobacco smoke exposure^3^. A review similarly describes biomass-associated COPD as commonly featuring cough/phlegm, airway thickening and air trapping, with emphysema and very low DLCO being less typical than in tobacco-related disease^10^. These findings, with the current cohort’s higher emphysema proportion in smoking-associated COPD and higher bronchiectasis rates in biomass and/or combined exposure, suggest a shift toward airway-dominant pathology when biomass exposure is present.

Exacerbations are central drivers of COPD burden and prognosis, and guideline frameworks increasingly emphasize future risk and disease activity^1^. Previous studies have reported that exacerbation rates do not consistently differ between biomass-associated COPD and tobacco smoke-associated COPD^8,20^. An analysis based on two prospective cohorts found no significant difference in the incidence of moderate-to-severe exacerbations between patients predominantly exposed to biomass smoke and those predominantly exposed to tobacco smoke (adjusted IRR=1.03; 95% CI: 0.56–1.89)^20^. In contrast, data focusing specifically on clinical outcomes in COPD with combined exposure remain limited, as many studies have defined phenotypes based on a single predominant exposure or have not sufficiently characterized dual exposure as a distinct ‘etiotype’. One study explicitly highlighted that the differential effect of exposure to tobacco, biomass, or both has not been adequately characterized^12^. Within this context, our study suggests that the combined exposure group may exhibit a worse disease trajectory. Combined exposure had the highest annual exacerbation rates, and also higher severe exacerbations and ICU and/or MV-requiring events. Chronic respiratory failure was also greatest in combined exposure, including higher LTOT use. Pulmonary hypertension was notably more common in combined exposure. Given that respiratory failure and pulmonary hypertension are clinically important complications of COPD, their higher frequency in the combined-exposure group may indicate a greater burden of adverse clinical outcomes. However, because of the observational design of this study, no causal or prognostic inference can be made. These findings suggest that patients with dual exposure to tobacco and biomass smoke may warrant careful clinical assessment and further investigation in prospective studies.

High bronchiectasis prevalence in the combined exposure group may provide an additional explanation for severe events. Bronchiectasis–COPD overlap is recognized as a clinically relevant overlap with recurrent exacerbations; when associated with infections, it impacts disease progression, exacerbations, and risk of death^21^. In a population-based cohort, bronchiectasis–COPD overlap was associated with substantially higher risks of acute respiratory events, including exacerbations and acute respiratory failure^22^. Similarly, Lahousse et al.^23^ reported that chronic bronchitis in COPD was associated with increased risk of exacerbations and mortality. The higher prevalence of bronchiectasis in the combined-exposure group was observed alongside a greater exacerbation burden; however, the observational design precludes causal interpretation.

Data comparing combined tobacco plus biomass smoke exposure with single exposures are limited; previous studies evaluating exacerbation burden across tobacco- and biomass-related COPD have yielded inconsistent findings. In a recent large real-world prospective study of 4436 patients, the combined cigarette- and biomass-exposure group had a higher incidence of moderate-to-severe exacerbations than either exposure group alone, which is consistent with the greater exacerbation burden observed in our combined-exposure group^24^. In contrast, Cho et al.^20^ found no significant difference in moderate-to-severe exacerbation rates across tobacco and biomass exposure strata, including patients with substantial exposure to both, while another study reported no difference in previous-year exacerbation frequency among tobacco-, biomass-, and dual-exposure COPD groups despite lower oxygen saturation in the dual-exposure group^12^. These discrepancies may reflect differences in sample size, exposure definitions and quantification, sex and age distributions, outcome ascertainment, covariate adjustment, and underlying comorbidity burden. In a comparative analysis of COPD patients exposed to tobacco smoke, biomass smoke, or both, Olloquequi et al.^12^ reported significant differences in clinical and functional parameters and systemic inflammatory patterns among groups, suggesting that combined exposures are not simply additive in a linear sense but may reflect distinct inflammatory or endotypic profiles. Direct evidence regarding pulmonary hypertension in dual-exposure COPD remains limited; however, a cross-sectional study by Shaik and Lawande^25^ similarly reported a higher frequency of pulmonary hypertension among patients exposed to both tobacco and biomass smoke than among those with either exposure alone. The greater burden of adverse outcomes observed in the combined-exposure group may indicate a different clinical profile; however, longitudinal studies are needed to determine its prognostic significance.

### Limitations

Several limitations warrant cautious interpretation of these findings. First, the retrospective design precludes causal inference and remains vulnerable to residual confounding. Unmeasured or incompletely captured variables such as socioeconomic status, co-exposures (e.g. ambient air pollution and passive smoking), early-life determinants, and disparities in access to healthcare may be differentially distributed across exposure groups and influence both exposure patterns and clinical outcomes. The retrospective design of the study also limits interpretation of temporal relationships. Although patients in the combined-exposure group were younger at the time of evaluation despite having a greater burden of selected adverse clinical outcomes, age at evaluation should not be interpreted as evidence of earlier COPD onset or accelerated disease progression because the temporal sequence between exposure, disease onset, and subsequent clinical deterioration was not directly assessed. Furthermore, residual confounding, exposure misclassification, differences in comorbidity burden, and coexisting airway disease, such as bronchiectasis, may have contributed to the observed associations. Therefore, our findings should be interpreted as exposure–outcome associations rather than evidence of a causal effect of combined exposure. Prospective longitudinal studies incorporating time-resolved quantitative exposure assessment, standardized exacerbation surveillance, and systematic evaluation of pulmonary hypertension are needed to determine whether dual exposure is independently associated with subsequent disease progression and adverse outcomes. Second, exposure misclassification is a potential concern, as biomass exposure was primarily reconstructed from patient history rather than objective personal monitoring. Consequently, commonly used metrics such as ‘years of exposure’ and ‘exposure hour-years’ may inadequately reflect true exposure burden, failing to account for critical dimensions including exposure intensity, fuel and stove characteristics, ventilation conditions, and temporal variability. Because exposure classification was based on retrospectively documented histories, some degree of diagnostic or etiological misclassification cannot be excluded, particularly in patients with incompletely characterized lifetime exposures. Third, thoracic CT was not systematically performed in all participants, introducing potential detection bias for radiological findings such as emphysema and bronchiectasis, which may have been more likely to be identified in patients who underwent CT imaging. In addition, pulmonary hypertension (PH) was not systematically evaluated for alternative etiologies, and right heart catheterization was not performed in the majority of patients. Given that PH in this cohort was predominantly considered to be Group 3 PH associated with COPD, invasive hemodynamic assessment was not routinely undertaken. Finally, because the study included stable COPD patients from two tertiary-care centers in one city, the findings may not be fully generalizable to primary-care populations, other geographical regions, or settings with different demographic characteristics, biomass-fuel practices, and healthcare access. Although the combined-exposure group included 80 patients, this number exceeded the prespecified minimum sample size of 69 patients per group derived from the *a priori* power analysis; however, larger studies may still improve the precision of estimates for less frequent outcomes.

## CONCLUSIONS

Findings of this study suggest that biomass-associated COPD is not a milder disease compared to smoking-related COPD; however, combined exposure appears to confer a significantly worse outcome. Combined cigarette and biomass smoke may cause earlier COPD presentation but worse disease impact: higher comorbidity burden, lower FEV_1_, and higher exacerbation rates. Future prospective longitudinal studies with larger, more diverse populations and standardized quantitative exposure assessment are needed to confirm these associations and clarify their temporal and clinical significance.

## Data Availability

The data supporting this research are available from the authors on reasonable request.
